# Optimization of Peripheral Blood Mononuclear Cell Extraction from Small Volume of Blood Samples: Potential Implications for Children-Related Diseases

**DOI:** 10.3390/mps5020020

**Published:** 2022-02-24

**Authors:** Deanira Patrone, Nicola Alessio, Nicola Antonucci, Anna Lisa Brigida, Gianfranco Peluso, Umberto Galderisi, Dario Siniscalco

**Affiliations:** 1 Department of Experimental Medicine, Division of Molecular Biology, Biotechnology and Histology, University of Campania “Luigi Vanvitelli”, via S. Maria di Costantinopoli 16, 80138 Naples, Italy; de.patrone@studenti.unina.it (D.P.); nicola.alessio@yahoo.it (N.A.); brigida.annalisa@gmail.com (A.L.B.); umberto.galderisi@unicampania.it (U.G.); 2 Biomedical Centre for Autism Research and Therapy, 70126 Bari, Italy; info@antonucci.eu; 3 Research Institute on Terrestrial Ecosystems (IRET), National Research Council of Italy (CNR), via P. Castellino 111, 80131 Naples, Italy; gianfranco.peluso@cnr.it; 4 Centre for Autism—La Forza del Silenzio, 81036 Caserta, Italy; 5 European Biomedical Research Institute of Salerno (EBRIS), 84125 Salerno, Italy

**Keywords:** peripheral blood mononuclear cells (PBMC), sample size, cellular extraction, children diseases

## Abstract

Managing medical procedures for children with problematic disorders is a challenging approach, especially in the case of blood withdrawal for autism spectrum disorder-affected children. Peripheral blood mononuclear cells (PBMC) represent an important cellular model to study immune responses and drug toxicity. The monocytic cells, a fraction of PBMC, are strongly involved in some pathophysiological processes, such as inflammation and immune system changes. Here, we propose a simple, reliable protocol for obtaining peripheral blood-derived mononuclear cells from small volumes of blood samples.

## 1. Introduction

Peripheral blood mononuclear cells (PBMC) represent an optimal source of cells for deep studies of several pathophysiological disorders. These cells comprise a heterogenous population of several round nuclei-cell types: monocytes, lymphocytes (B-cells, T-cells) and Natural Killer (NK) cells; differentiated dendritic and macrophagic cells, as well as stem cells, can be also present. The specific cellular sub-type composition of PBMC depends on the donor and is based on the donor health status; typically in humans, lymphocytes are most abundant, ranging from 70 to 90%, then monocytes are present in 10–20%, and other cells, including dendritic cells, are for only 1–2% [1]. All these cells belong to the innate and adaptive immune systems, and are responsive against viral, bacterial, and parasitic infections. Despite the limited cell lifespan on in vitro culture, their use as cellular model is widespread. In addition, these cells represent an optimal model for studying toxicity and immunosuppressive drugs [2], as well as for environmental exposure analysis. Furthermore, PBMC are also used for in vivo cell transplantation [3]. Among PBMC subsets, monocytes are the optimal candidate for studying inflammatory states and altered immune responses [4]. The main advantage lies in their easy and almost unlimited source. However, blood withdrawal could be difficult if the donors are children [5]; in particular in the case of the presence of problematic disorders, such as autism spectrum disorder (ASD). The required small size of blood samples is a concern for the yield, also affecting purity and reproducibility. Usually, PBMC are extracted by ficoll-gradient protocol; here, we establish a simple and reliable protocol for PBMC extraction from very small sample sizes.

## 2. Experimental Design

### 2.1. Materials

- Histopaque 1077 density gradient (Sigma Aldrich, St Louis, MO, USA; cat. n. 10771) orLymphocyte Separation Medium (Lonza, Walkersville, MD, USA; cat. n. 17-829E), or Lymphosep (Microgem, Naples, Italy; cat. n. L0560).
- Dulbecco’s Phosphate buffered saline D-PBS (Sigma-Aldrich, St. Louis, MO, USA; cat. n. D8662).
- Trypan blue (Sigma Aldrich, St Louis, MO, USA; cat. n. T8154).
- Ammonium-Chloride-Potassium (ACK) lysing buffer (Thermo Fisher, Waltham, MA, USA; cat. n. A1049201).

### 2.2. Equipment

- EDTA tubes (Fisher Scientific, Pittsburgh, PA, USA; model BD 367862).
- 2 mL and 5 mL sterile and disposable serological pipettes (BD Falcon Becton Dickinson Labware, Franklin Lakes, NJ, USA; cat. n. 357507 and n. 357543, respectively).
- 15 mL conical, sterile, polypropylene, centrifuge tubes (Fisher Scientific, Pittsburgh, PA, USA; model Corning 352196).
- Pipettes (Eppendorf Research, Milan, Italy; cat. n. 3123000020 (0.5–10 μL), n. 3123000047 (10–100 μL), n. 3123000063 (100–1000 μL), with relative sterile tips).
- Electronic pipette (Eppendorf Research, Milan, Italy; cat. n. 4430000018).
- Pasteur pipette.
- 1.5 mL tube (Eppendorf Research, Milan, Italy; cat. n. 0030125150).
- Burker cell counter (Sigma-Aldrich, St. Louis, MO, USA; cat. n. Z359629) and coverslip (Sigma-Aldrich, St. Louis, MO, USA; cat. n. Z375357).
- Centrifuge (Thermo Scientific, Cincinnati, OH, USA; model Heraeus Megafuge 1.0).
- Class II biological safety cabinet (Steril, Lecce, Italy; model. n. VBH 48 MP VBH).
- Inverted microscope (Leica Microsystems, Milan, Italy; model DMi1).
- Humidified 95% O_2_/5% CO_2_ water jacketed incubator, 37 °C (Thermo Scientific, Cincinnati, OH, USA; model Forma Series II) (optional).
- Flow Cytometer (Merck Millipore, Burlington, MA, USA; model Guava^®^ easyCyte 5 HPL Benchtop Flow Cytometer).

## 3. Procedure

**Figure 1:**
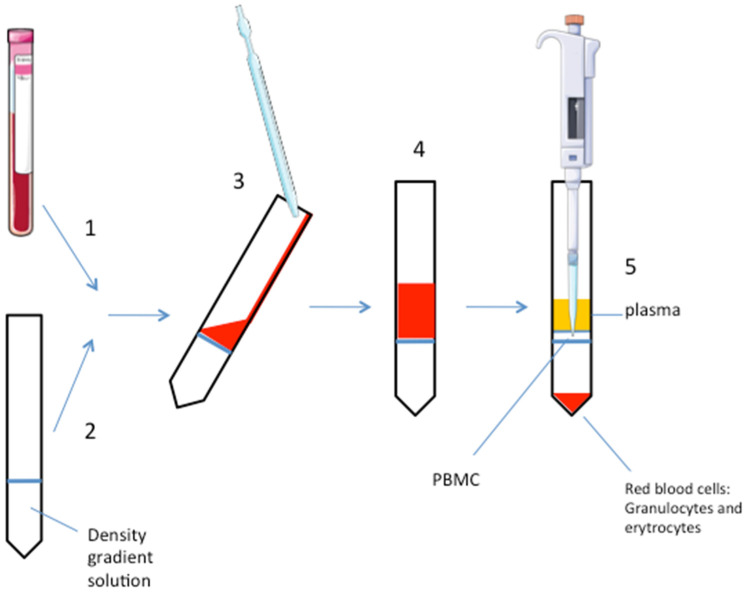
Schematic representation of the procedure. Numbers refer to the procedure steps in the text. The pipettes and the EDTA-tube are drawn using pictures from Servier Medical Art https://smart.servier.com/ under a Creative Commons Attribution 3.0 Unported License.

All the steps of the experiment are conducted in sterile conditions. See Figure 1 for a schematic representation.

(1) Collect the blood sample in an EDTA-tube (volume: 2 mL).
(2) Add the density gradient solution (i.e., polysucrose, ρ = 1.077 g/mL, or the highly branched copolymer of sucrose and epichlorohydin monomers: ficoll) to a clean 15 mL tube in a volume 1:1 to the blood sample. Critical step: do not dilute blood with D-PBS to avoid too much dilution of the small sample.
(3) Carefully and slowly, add the blood sample on the gradient solution without mixing them to ensure a defined interphase separation. This step should be done by inclining the tube and adding the blood sample very slowly. Using the wall of the tube to help, pipet down the blood on the gradient solution.
(4) Centrifuge at 1010× *g* for 30 min at room temperature. Do not use the rotor brake for avoiding phases mixing. When the rotor is totally stopped, carefully remove the tube, paying attention to not mix the phases.
(5) After centrifugation, PBMC are stratified on the middle phase (cloudy white interface). At the top of the tube, there is the plasma, erytrocytes and granulocytes are stratified to the bottom. A well-defined interface is a marker of an optimal phase separation. Carefully aspirate the PBMC phase with a pipette and put it into a clean 15 mL tube. Another way is to aspirate and discard the upper plasma phase before pipetting the PBMC layer. Consider that the gradient solution could be toxic for the cells, so avoid too much time for this procedure step, if the experiment requires growing the PBMC in culture at the end of the extraction.
(6) Add D-PBS in 1:1 in volume. Gently mix.
(7) Centrifuge at 605× *g* for 10 min at room temperature with rotor brake on.
(8) Remove and discard supernatant and add D-PBS, typically the volume ranges from 1 mL to 3 mL, depending on the pellet size; carefully re-suspend the pellet.
(9) Centrifuge at 300× *g* for 10 min at room temperature with rotor brake on.
(10) Remove and discard supernatant and re-suspend cells in the desired buffer (i.e., growing media, lysis buffer, or others).
(11) Count cells with Trypan blue; or see next step (optional).
(12) Acquire the sample on standard or multicolor flow cytometer and analyze it following a standard procedure (optional).

## 4. Expected Results

We already successfully demonstrated PBMC extraction from a little sample size volume (10 mL) from autistic children [6]. Here, we propose an easily method to further decrease the initial blood volume required (2 mL). Obtained PBMC can be used for several downstream cellular and molecular analysis.

Our expected yield we obtained (as mean ± standard deviation) is 2 × 10^6^ ± 3 × 10^5^ cells/mL of initial blood. The Pearson’s range value we obtained, as a measure of dispersion, a measure of how much each individual data can differ from their average, is 8 × 10^5^. We used the following formula to calculate the range: Range = maximum (x_i_) − minimum (x_i_), where x_i_ represents the set of values. This value indicates a good dispersion index.

**Figure 2:**
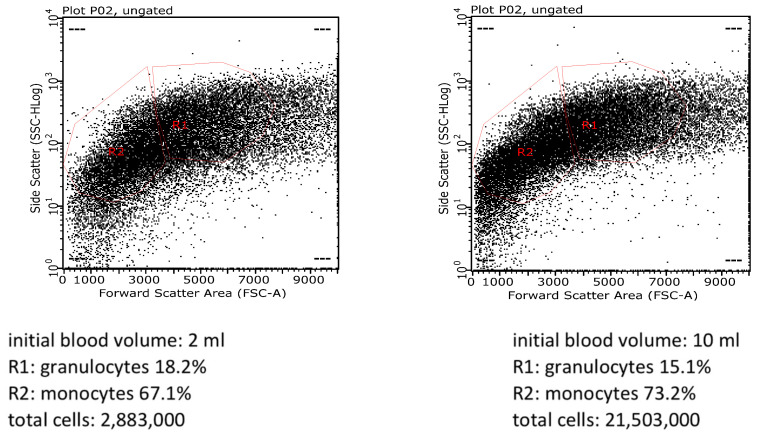
Flow cytometry analysis. (**Left**) Representative plot from 2 mL of initial blood volume (our new protocol); (**Right**) representative plot from 10 mL of initial blood volume (standard technique). (Data were acquired using the flow cytometer model Guava^®^ easyCyte 5 HPL Benchtop Flow Cytometer (Merck Millipore, Burlington, MA, USA).

**Figure 3:**
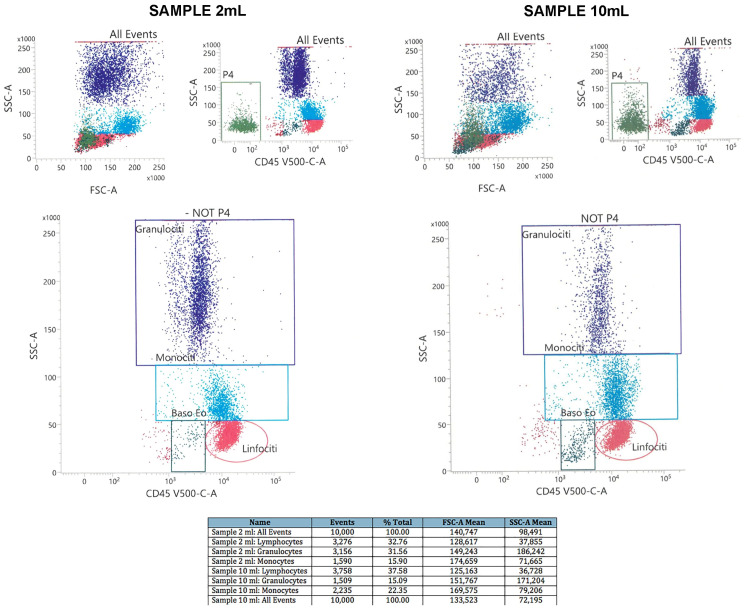
Flow cytometry analysis. (**Left**) Representative plot from 2 mL of initial blood volume (our new protocol); (**Right**) representative plot from 10 mL of initial blood volume (standard technique). Legend of the cell names: *Granulociti*: granulocytes; *Monociti*: monocytes; *Linfociti*: lymphocytes. FSC-A: forward scatter area. SSC-A: side scatter area. The table reports the quantification of the cell subtypes for both the sample sizes.

We also performed flow cytometry analysis to further compare our protocol versus standard technique. Figure 2 shows the plots of scatter parameters providing information about the fraction of cell subtypes we obtained with our method versus the standard one. We obtained almost 3 × 10^6^, whereas from 10 mL of initial blood sample, our yield was 21 × 10^6^ cells. Notably, with our protocol, in the cellular population we identified 67.1% of monocytes and 18.2% of granulocytes, compared to 73.2% of monocytes and 15.1% of granulocytes we obtained with the standard procedure. This result further confirms the validity of our new method. Figure 3 shows additional plots of scatter parameters using multicolor flow cytometry, allowing a better characterization of cell numbers and subtypes we obtained, with comparison between 2 mL^−^ vs. 10 mL^−^ of initial blood samples. In this case, from 2 mL of initial blood samples, we identified 15.9% of monocytes, 32.8% of lymphocytes and 32.8% of granulocytes, compared to 22.3% of monocytes, 37.6% of lymphocytes and 15.1% of granulocytes we obtained from 10 mL of the initial blood. These results further confirm the reliability of our protocol. 

Expected yield in the pediatric population (2–5 years) ranges from 1 to 6 × 10^6^ cells/mL [7]. However, according to [7], the small volume blood withdrawal for sample collection for adolescent/pediatric subject is 10 mL, with an average of cellular yield of 1.5 × 10^6^ cells/mL. Here, we demonstrate how to obtain the same yield in terms of cell/mL starting from a smaller blood volume. Specifically, we modified the standard preparation method in several steps: small volume required (the standard procedure requires at least 10 mL of blood); in avoiding blood dilution with D-PBS to avoid too much dispersion of the sample; in the centrifugation time and g; and in the washing steps. With these few but key modifications to the existing protocols [8], this procedure could be very helpful for managing children under stress, as in the case of blood withdrawal for autistic patients. Indeed, ASD children require additional health care and specialized services during their hospitalization [9]; decreasing the procedure time for blood withdrawal, as well as the blood volume required, will contribute to lowering the potentially challenging behaviors. Finally, a small volume of samples can be obtained from all children. We emphasized ASD children due to their particular behavioral assessment. However, the small volumes could be also applied to adults.

### Troubleshooting

Cell yield dramatically depends on the donor’s status. Healthy subjects give better results. Pediatric populations typically show higher lymphocyte counts than adults, hence the yield is increased. However, lymphocytes (non-adherent cells) can be easily removed from the culture to obtain purified monocyte population [6].

EDTA, used as an anti-coagulant in tubes for blood collection, could affect the yield over time. It is critical to use fresh blood samples (from 1 h after withdrawal); if it is not possible to achieve this step, blood storage should be performed at room temperature, under agitation, at maximum for 24 h, as cells die over time. Longer storage (more than 24 h) decreases cell recovery and viability [10,11]. Inspection of the sample into the tube in order to exclude blood coagulation is needed. Room temperature also affects purity: warmer temperatures increase red blood cell contamination. In order to eliminate eventual contaminating red blood cells (RBC), PBMC pellet could be re-suspended in an ammonium-chloride-potassium (ACK) lysing buffer for 10 min at room temperature [12]. Removing RBC could be useful for following flow cytometry analysis. If the aim of PBMC extraction is to grow monocytes on in vitro culture, eventual contaminating RBC can be easily removed, as non-adherent cells, from the dish. If the aim is to perform gene expression analysis, it is noteworthy to consider that RBC are without nuclei. For flow cytometry analysis, no lysis no wash (NLNW) protocols have seen a limited use [13]. However, the lysis methods could cause changes in differential white blood cell scatter profiles [13]. The NLNW advantage is avoiding cell loss during washing and reducing sample manipulation and related artifacts; whereas the main disadvantage is lower throughput, due to the several dilutions needed to reduce the non-specific fluorescence backgrounds [13].

## Data Availability

The data presented in this study are available within the article.

## References

[1] Kleiveland C.R., Verhoeckx K., Cotter P., López-Expósito I., Kleiveland C., Lea T., Mackie A. (2015). Peripheral Blood Mononuclear Cells. The Impact of Food Bioactives on Health.

[2] Pourahmad J., Salimi A. (2015). Isolated Human Peripheral Blood Mononuclear Cell (PBMC), a Cost Effective Tool for Predicting Immunosuppressive Effects of Drugs and Xenobiotics. Iran. J. Pharm. Res..

[3] Yang D.N., Wu J.H., Geng L., Cao L.J., Zhang Q.J., Luo J.Q., Kallen A., Hou Z.H., Qian W.P., Shi Y. (2020). Efficacy of intrauterine perfusion of peripheral blood mononuclear cells (PBMC) for infertile women before embryo transfer: Meta-analysis. J. Obstet. Gynaecol..

[4] Siniscalco D., Sapone A., Giordano C., Cirillo A., De Magistris L., Rossi F., Fasano A., Bradstreet J.J., Maione S., Antonucci N. (2013). Cannabinoid Receptor Type 2, but not Type 1, is Up-Regulated in Peripheral Blood Mononuclear Cells of Children Affected by Autistic Disorders. J. Autism Dev. Disord..

[5] Cuper N.J., Klaessens J., Jaspers J.E., De Roode R., Noordmans H.J., De Graaff J., Verdaasdonk R.M. (2013). The use of near-infrared light for safe and effective visualization of subsurface blood vessels to facilitate blood withdrawal in children. Med. Eng. Phys..

[6] Siniscalco D., Bradstreet J.J., Cirillo A., Antonucci N. (2014). The in vitro GcMAF effects on endocannabinoid system transcriptionomics, receptor formation, and cell activity of autism-derived macrophages. J. Neuroinflamm..

[7] (2018). HIV/AIDS Network Coordination: Cross-Network PBMC Processing Standard Operating Procedure. www.hanc.info.

[8] Böyum A. (1968). Isolation of mononuclear cells and granulocytes from human blood. Isolation of monuclear cells by one centrifugation, and of granulocytes by combining centrifugation and sedimentation at 1 g. Scand. J. Clin. Lab. Investig. Suppl..

[9] Taghizadeh N., Heard G., Davidson A., Williams K., Story D. (2019). The experiences of children with autism spectrum disorder, their caregivers and health care providers during day procedure: A mixed methods study. Pediatr. Anesth..

[10] Olson W.C., Smolkin M.E., Farris E.M., Fink R.J., Czarkowski A.R., Fink J.H., Chianese-Bullock K.A., Slingluff C.L. (2011). Shipping blood to a central laboratory in multicenter clinical trials: Effect of ambient temperature on specimen temperature, and effects of temperature on mononuclear cell yield, viability and immunologic function. J. Transl. Med..

[11] Palmirotta R., De Marchis M.L., Ludovici G., Leone B., Savonarola A., Ialongo C., Spila A., De Angelis F., Ferroni P., Della-Morte D. (2012). Impact of preanalytical handling and timing for peripheral blood mononuclear cells isolation and RNA studies: The experience of the Interinstitutional Multidisciplinary BioBank (BioBIM). Int. J. Biol. Markers.

[12] Corkum C.P., Ings D.P., Burgess C., Karwowska S., Kroll W., Michalak T.I. (2015). Immune cell subsets and their gene expression profiles from human PBMC isolated by Vacutainer Cell Preparation Tube (CPT™) and standard density gradient. BMC Immunol..

[13] Petriz J., Bradford J.A., Ward M.D. (2018). No lyse no wash flow cytometry for maximizing minimal sample preparation. Methods.

